# The Teaching of Physiology

**Published:** 1920-09-11

**Authors:** 


### THE TEACHING OF PHYSIOLOGY.

A SINGULARLY important point in connection with the generally accepted teaching methods in our medical schools was raised by Dr. T. Lewis, F.R.S., at the recent meeting of the British Association, and although this reference had perhaps been most aptly made in our Educational Number of last week yet pressure on our available space made postponement essential. The speaker's attitude was mainly that of a physician; leaving aside the obvious, if minor, differences of outlook which will always exist between the clinicians and the pure scientists, we may with advantage follow his line of argument as throwing considerable light upon one of the great teaching fallacies of the time. We admit to having seen active steps towards reform being taken in many quarters, but there is undoubtedly danger of falling short of the reconstructional ideal if the truth is not driven thoroughly home.

Physiology, although undoubtedly an offspring of the medicine of early times, has now come to be to the healing profession a chief pillar of support. Inevitably, much experimental physiological work must be performed and demonstrated upon the lower animals, but are we quite sure that during the last few years we have not overdone the teaching of animal physiology as such? In the end, at the time of a man's final training the study of the human as opposed to the animal must form the central point of his perfected education. Dr. Lewis admits that it is impossible to confine physiological lessons to the human body since by far the largest part of our knowledge of the functions of the human body is still derived from analogical observations on the beast. But knowledge that has been obtained and is still being obtained from observation of man himself should be constantly stressed from the very earliest parts of the medical curriculum, and the whole teaching of the medical student so turned that the general sciences, among which we may justifiably include the study of animal bodily functions, should be given from the outset their true secondary position as merely leading up to and amplifying our knowledge of human existence. It is of considerably greater importance that the student should witness a normal phenomenon, or understand a given principle, by observation on the human rather than the animal system, and it is only where this cannot be satisfactorily brought about, not at least with a satisfactory degree of certainty, that pure animal physiology should be called in. This should undoubtedly be the trend of modern teaching of a most vital subject and, as we admit, there is evidence that many of those responsible are beginning to realise the urgency. A few more insistent demonstrations from men of great experience should do much to ensure the continuance of a very necessary reform.

But there is another method of achieving this end and one which is singularly efficacious in teaching circles as it affects with great directness both student and tutor. This is the attitude of the various examining bodies and the type of knowledge and the state of mind which examiners demand in the men considered to have performed satisfactorily the earlier part of their medical training. Here we withhold our criticism of the standard often considered as satisfactory in the young student about to enter the wards of a hospital and actually to start his first ministrations to the sick, but we think there is strong justification for objection to the amount of concentration on purely animal physiology which many examiners apparently require. It is all very well to know how to dissect out and stimulate the vagus nerve in the frog and demonstrate the result satisfactorily in the examintion room, but how often can the same student at this stage demonstrate that pressure upon the vagus in the carotid sheath of a normal man will provide a similar picture of lapses in the heart's beat?

In any circumstances, we have to face the fact that medical students will ultimately have to meet physiological problems far more complicated than those of their animal experiment days. The bedside analogue of every one of their early experiments must be explained to them at the same time, and until physiological teachers carry out this principle as far as it is in their power we shall always meet that state of mind in the student which Dr. Lewis so greatly deplores. "Few of those when they come to the teaching hospitals from the laboratories have, in even the broadest sense of the term, learned to examine the bodily functions of a human being, their senses of touch and hearing are, in a professional sense, utterly undeveloped, and yet they are expected to handle men for the rest of their days." The first step towards remedy appears to us to lie in a greatly refined teaching of physiology in the intermediate stages of the medical education of the student.

